# Dr. James Albert Oliver

**Published:** 1908-01

**Authors:** 


Dr. James Albert Oliver, of Steubenville, O., died at his home from erysipelas, December 1, 1907, aged 55 years. He graduated from the medical department of Niagara University in 1896.

